# The processive kinetics of gene conversion in bacteria

**DOI:** 10.1111/mmi.13661

**Published:** 2017-03-13

**Authors:** Johan Paulsson, Meriem El Karoui, Monica Lindell, Diarmaid Hughes

**Affiliations:** ^1^ Department of Systems BiologyHarvard UniversityBostonMA02115USA; ^2^ School of Biological SciencesInstitute of Cell Biology, The University of EdinburghEdinburghEH9 3JRUK; ^3^ Department of Medical Biochemistry and MicrobiologyBox 582, Biomedical Center, Uppsala UniversityUppsalaS‐751 23Sweden

## Abstract

Gene conversion, non‐reciprocal transfer from one homologous sequence to another, is a major force in evolutionary dynamics, promoting co‐evolution in gene families and maintaining similarities between repeated genes. However, the properties of the transfer – where it initiates, how far it proceeds and how the resulting conversion tracts are affected by mismatch repair – are not well understood. Here, we use the duplicate *tuf* genes in *Salmonella* as a quantitatively tractable model system for gene conversion. We selected for conversion in multiple different positions of *tuf*, and examined the resulting distributions of conversion tracts in mismatch repair‐deficient and mismatch repair‐proficient strains. A simple stochastic model accounting for the essential steps of conversion showed excellent agreement with the data for all selection points using the same value of the conversion processivity, which is the only kinetic parameter of the model. The analysis suggests that gene conversion effectively initiates uniformly at any position within a *tuf* gene, and proceeds with an effectively uniform conversion processivity in either direction limited by the bounds of the gene.

## Introduction

Gene conversion is a recombination phenomenon in which genetic information is transferred non‐reciprocally between almost identical sequences. It occurs between homologous chromosomes as well as between repeated sequences on the same chromosome (Jackson and Fink, 1981), and plays a key evolutionary role by transferring mutations between members of gene families. Specifically, conversion can benefit cells by accelerating the spread of advantageous mutations or reverting multiple slightly deleterious mutations in a single event. Gene conversion could act to keep repeated genes close to identical, but gene conversion also plays an important role in e.g. the male‐specific testis regions of the human Y chromosome (Rozen *et al*., 2003), mating‐type switching in budding yeast (Haber, 1998), pilin antigenic variation (Cahoon and Seifert, 2009) and the acquisition of antibiotic resistance in pathogenic bacteria (Miller *et al*., 2008).

**Figure 1:**
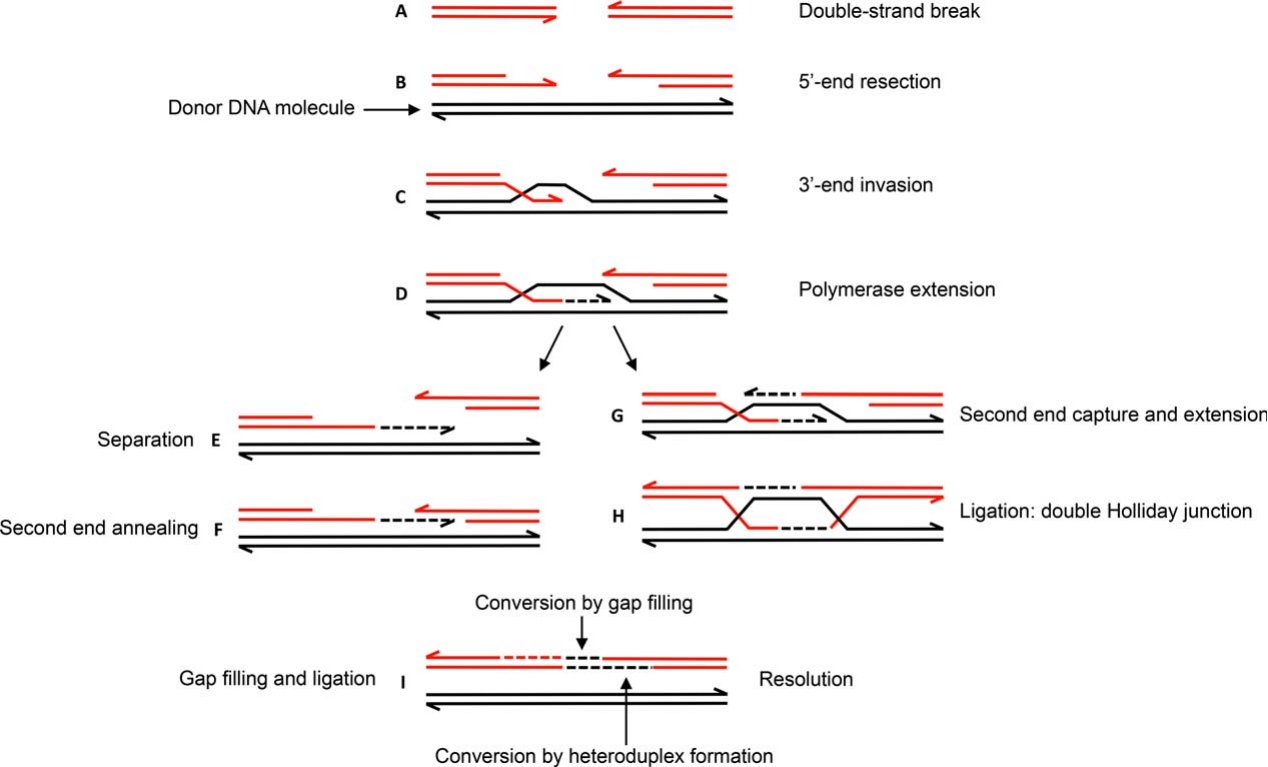
Basic model for gene conversion. The directional process is indicated by the letters A–I. The process is initiated by a DSB in one gene (Hastings, 2010). This is processed by RecBCD exonuclease/helicase to create a gap and an ssDNA (arrow heads are 3′‐ends). RecA promotes strand invasion of an undamaged homologous (almost identical) gene (thin vertical lines represent mismatches in heteroduplex DNA, hDNA). The invading tail then initiates new DNA synthesis which extends until it overlaps and anneals with the other end of the DSB. Through a similar process, the displaced DNA strand forms hDNA with the second 3′‐end ssDNA. Two Holliday Junctions are formed which are able to migrate and extend hDNA. Segments of gene conversion can be generated in two different ways. One alternative is through repair, by DNA synthesis, of the gap produced during initiation. The other is through the MMR system which may “repair” hDNA by destroying one strand of DNA in the heteroduplex, followed by new DNA synthesis using the remaining strand as template.

These observations have motivated detailed genetic and biochemical studies of the underlying mechanisms of gene conversion. The most widely accepted molecular model focuses on double‐strand‐break (DSB) repair (Orr‐Weaver *et al*., 1988; Hastings, 2010), where gene conversion is initiated at a DSB and exonucleases extend the gap to allow invasion of a homologous sequence (Fig. 1). Holliday junctions (HJ) are then formed and migrate along the gene until they are resolved by cutting DNA, terminating the process. Because the sequences involved are not strictly identical, and some of the mismatches escape the methyl‐directed mismatch repair system (MMR), the result is a transfer of genetic information from an unbroken template region to the broken one, usually in continuous blocks termed gene conversion tracts. The key molecular players have been well characterized in *Escherichia coli* and *Salmonella enterica*. However, little is known quantitatively about the process, for example whether it initiates and terminates at random nucleotides or only at specific hotspots, how conversion tracts are extended in both directions, what happens if the process reaches the ends of the homologous sequences, and to what extent conversion is influenced by other components and events. Because conversion occurs briefly once every 10^5^−10^6^ generations, these questions cannot easily be addressed either in bulk averages or by monitoring the process in real time using fluorescent probes in single cells. Many previous studies have estimated recombination rates and the sizes of recombination tracts in bacteria, archaea and fungi (Feil *et al*., 2000; Falush *et al*., 2001; Vos and Didelot, 2009; Didelot *et al*., 2011; Croucher *et al*., 2012; Everitt *et al*., 2014; Mell *et al*., 2014; Bubendorfer *et al*., 2016). However, these studies have focused on horizontally transferred genetic material, where sequence identities supporting homologous recombination can extend far beyond the boundaries of a gene. In this work we have focussed on non‐reciprocal gene conversion events involving two paralogous genes within the same chromosome and bacterial cell, and where the region of sequence identity ends at the boundaries of the respective coding sequences (Abdulkarim and Hughes, 1996).

Here, we translate the molecular DSB repair model of gene conversion into a simple stochastic reaction model, to predict how fluctuations in conversion tract lengths depend on the underlying mechanisms and the length of the repeated regions. This allows us to experimentally test assumptions about the process against distributions of the final conversion tracts, without real‐time observations. Such analyses are still challenging because few cells undergo gene conversion, because sequence homology hides the extent of conversion, and because MMR both reduces the observed conversion frequencies and changes the shape of the conversion tract distributions. However, using the two nearly identical genes *tufA* and *tufB* in the chromosome of *S. enterica* as a model system allows us to work around these limitations, and measure the conversion tracts of individual cells in both MMR deficient and MMR proficient strains.

## Results and discussion

### Experimental assay to measure individual conversion tracts

**Figure 2:**
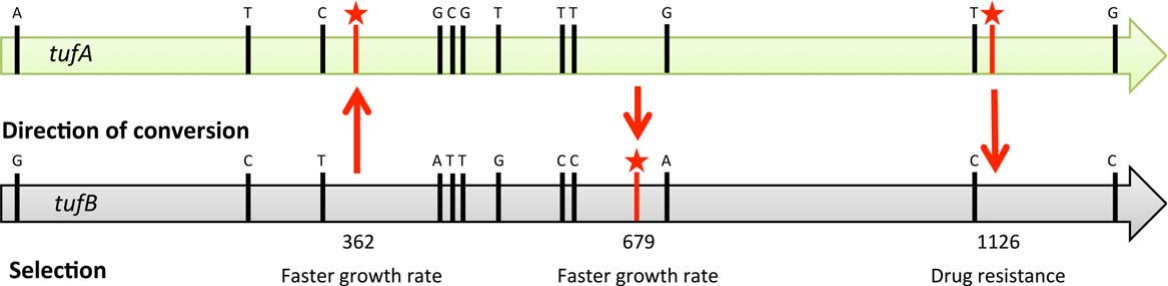
Selection scheme for gene conversion involving *tufA* and *tufB*. Vertical black bars indicate the locations of the 12 nucleotide differences between the *tufA* and *tufB* genes. Vertical red bars with a star indicate the locations of mutations used in the selection of recombinants in different strains. The mutations at nt 362 (CTG Leu to CAG Gln) and nt 679 (GTT Val to TTT Phe) each cause very slow growth and selection for faster growing colonies selected for recombinants that had undergone gene conversion, in each case including repair of the mutation that caused slow growth. The mutation at nt 1126 (GCA Ala to ACA Thr) is recessive for drug‐resistance. Selection for drug‐resistance (kirromycin) selected for recombinants that carried this recessive mutation in both *tuf* genes (expressing resistance). The red arrows indicate the direction of genetic information transfer in each selection.

The bacterium *S. enterica* has two nearly identical and highly expressed genes *tufA* and *tufB* for the elongation factor EF‐Tu that delivers tRNAs to the ribosome (Tubulekas and Hughes, 1993; Brandis and Hughes, 2016). The genes are 1185 nucleotides (nts) long, are separated by 762 kbs in inverse orientations on the circular chromosome, and differ from each other at 12 dispersed single nucleotide positions (McClelland *et al*., 2001). By identifying cells in which conversion events occurred, sequencing both genes, and observing which of the 12 positions converted, it is thus possible to determine narrow intervals for the possible start and end points of each individual conversion tract (Fig. 2). The *tuf* system is particularly powerful for studying gene conversion (Abdulkarim and Hughes, 1996; Hughes, 2000; Arwidsson and Hughes, 2004) because mutations at distinct positions within *tuf* (nts 362, 679 and 1126) can be used to select bacteria in which recombination between the *tuf* genes has occurred that resulted in a gene conversion event that included the selected nucleotide (see *Experimental procedures*). This allows us to identify cells in which conversion occurred simply by screening colony growth on petri dishes and confirming the event by DNA sequencing. Furthermore, selecting for conversion at three different positions in the *tuf* gene, close to the beginning, middle and end of the gene, allows us to estimate three different conditional conversion tract distributions. This enables us to test mathematical models much more thoroughly than with a single selection point. Finally, by performing the experiments both for strains that are deficient and proficient in mismatch repair it is possible to analyze the conversion processes with and without subsequent modifications.

The actual experiments were very straightforward. Each evaluated strain was grown in multiple independent cultures, and each such culture was plated on a separate petri dish to screen or select for colonies where conversion occurred (Fig. 2). Both of the *tuf* genes were then sequenced to determine the length of the conversion tracts, and the whole procedure was repeated many times to statistically sample distributions and frequencies. We confirmed by sequencing that the selected position in the recipient gene was converted every time, and found that it was typically associated with the conversion of other, unselected, nucleotides, as expected for conversion. One technical question is whether these recombination events are conversion events in the strict sense of a unidirectional transfer of genetic information, or whether they could be examples of reciprocal recombination involving different sister chromosomes within the bacterial cell. Several lines of evidence support the interpretation that we are measuring unidirectional gene conversion events. First, we confirmed in every case, that only the recipient *tuf* gene and not the donor *tuf* gene acquired any nucleotide changes, and that these changes were always conversions to the sequence of the donor *tuf* gene. Second, we have shown previously that almost half of the conversion events selected at nt 1126 involve the simultaneous formation of a chromosomal inversion of the region between the *tuf* genes, as expected for recombination between two inversely oriented *tuf* genes located in the same chromosome, showing that the recombination has occurred between sequences on the same chromosome (Hughes, 2000). Third, in fluctuation tests, a jackpot analysis of the products of *tuf* gene conversion, using a genetic selection that could isolate and identify each of the expected products of reciprocal recombination between the *tuf* genes (Arwidsson and Hughes, 2004) found no evidence to support reciprocal recombination as an explanation for *tuf* conversion. These data and observations give us confidence that the events we are selecting and quantifying are gene conversion events involving the non‐reciprocal transfer of genetic information from one *tuf* gene to the other, on the same chromosome. Furthermore, more than 96% of the conversion tracts were contiguous: when two positions were converted, typically all positions between them were also converted. The rare non‐contiguous tracts may occur by a separate conversion mechanism or perhaps MMR‐independent repair, but their frequency is so low that it has an insignificant effect on the frequencies and distributions. Measured conversion frequencies are reported in Table 1.

**Table 1:** Strain genotypes and gene conversion rates.

		Gene conversion data				
Strain	Genotype	Nt^a^	Rate^b^	N^c^	Nc^d^	Comment
TH4498	*tufA*474 (L120Q) *tufB*442::*Mu*dJ (nt −17) *trpE*91	362	2 × 10^−6^	71	1	Repair‐proficient, full length
TH5037	*tufA*474 *tufB*442::*Mu*dJ (nt −17) *trpE*91 *mutS*121::*Tn*10	362	1 × 10^−3^	32	3	Repair‐deficient, full length
TH1193	*tufA*474 *tufB*441::*Mu*dJ (nt 713) *trpE*91	362	2 × 10^−7^	15	0	Repair‐proficient, truncated
TH7511	*tufA*474 *tufB*441::*Mu*dJ (nt 713) *trpE*91 *mutS*121::*Tn*10	362	1 × 10^−4^	40	0	Repair‐deficient, truncated
TH522	*tufA*8 (A375T) *tufB*430 (V226F) *trpE*91 *hisG*3720	679	1 × 10^−5^	85	0	Repair‐proficient, full length
TH5038	*tufA*8 *tufB*430 (V226F) *trpE*91 *hisG*3720 *mutS*121::*Tn*10	679	2 × 10^−3^	53	5	Repair‐deficient, full length
TH488	*tufA*8 *trpE*91 *hisG*3720	1126	2 × 10^−8^	37	3	Repair‐proficient, full length
TH3262	*tufA*8 *trpE*91 *hisG*3720 *mutS*121::*Tn*10	1126	2 × 10^−5^	61	2	Repair‐deficient, full length

**a.** Nucleotide position in *tuf* selected for conversion.

**b.** Conversion rate per cell per generation calculated from fluctuation tests.

**c.** Number of independent cultures measured for gene conversion rate.

**d.** Number of non‐contiguous gene conversion tracts observed.

### Modelling the kinetics of gene conversion as a stochastic process

We next model gene conversion using the DSB repair model framework in the absence of MMR (Supporting Information, S1 text, and Fig. 1). In the model, gene conversion is initiated by a random DSB in one copy of the repeated gene, creating two double strand ends, each of which can be processed to create a gap and a single strand DNA end (ssDNA). Both ssDNA ends can independently invade the uncut almost identical gene, which is used as a template to copy the missing genetic information. This results in conversion of the gene, or part of the gene (Fig. 1). The length of the conversion tracts depends on the size of the initial gap as well as the extension of the subsequent steps such as HJ migration (Santoyo and Romero, 2005). Since homologous recombination depends on sequence identity, we assume it cannot extend beyond the boundaries of the repeated genes. Translating these molecular observations into simple quantitative assumptions, we assume that:

1. A DSB occurs with equal probability at any position *i* within a gene of length *N*.
2. Starting from the initial DSB, gap formation and HJ migration extend the conversion tract independently in both directions, converting the next nucleotide with probability ρ and stopping with probability 1−ρ. Parameter ρ can thus be interpreted as an effective processivity parameter (i.e., the parameter measures the capacity of the process to act repeatedly on the same substrate without disengagement), and determines the length of the contiguous conversion tracts.
3. If the extensions proceed to either end of the gene, the whole event is aborted because of lack of sequence identity, and the outcome is not observed.

This model is an abstract and highly simplified representation of the molecular process – for example we do not know if local sequence effects will have a marginal impact on the three steps above. However, our aim here is to quantify the most important steps and avoid adding too many details: if several free parameters had to be tuned to achieve a fit, the analysis would hardly be conclusive because virtually any observations can be accommodated by adding *ad hoc* assumptions.

The assumptions above allow us to analytically calculate (Supporting Information) the probability *P*_eff_(*m*) that position *m* is successfully converted, given that there has been a DSB somewhere in the gene, as a function of the processivity parameter and the length of the gene:

(1) $$P_{\text{eff}}(m)=\frac{\rho^{m}-\rho^{N+1}}{N}\left(\frac{\rho^{-m}-1}{1-\rho }-m\right)+\frac{\rho^{-m}-1}{N}\left[\frac{\rho }{1-\rho }(\rho^{m}-\rho^{N})-\rho^{N+1}(N-m)\right]$$

Experimentally we measure gene conversion tracts by first selecting for conversion at some given position *n* (see above). To compare experiments and model we must thus calculate the conditional probabilities P(m|n) that the conversion tract contains position *m* given that it also contains position *n*. This follows:

(2) $$P(m|n)=\frac{(m-g)\rho^{-m}+g}{(n-g)\rho^{-n}+g}\;\mathrm{where}\;g(n)=\frac{1+\rho }{1-\rho }+n-\frac{N-n}{\rho^{n-N}-1}$$

for m<n while for m>n, symmetry produces the same expression with *m* exchanged for *N* − *m*. The length of the gene *N* and the selection points *n* are known exactly from experiments, leaving ρ as the single free model parameter.

Because gap formation and HJ migration are thought to on average extend hundreds or even thousands of nucleotides (Dillingham and Kowalczykowski, 2008; Rasnik *et al*., 2008), we also derive the limit distribution as ρ→1, for which the process almost always extends to the ends of the gene. It may then seem that the few events that do not extend all the way to the end of the gene still come close to the end, but this intuition is misleading and the process instead creates a nontrivial limit distribution:

(3) $$\underset{\rho \to 1}{\lim}\mathrm{Pr}(m|n)=\frac{m(N+n-m)}{nN}$$

where again *m* should be exchanged for *N* – *m* when *m* > *n*. The intuitive reason is that given that the process did not extend to either gene end, there are still many similarly probable positions to terminate within the gene. For gene lengths of *N* ≈ 1000 nucleotides, values of ρ>0.999 are virtually indistinguishable from ρ→1. The model then has *zero* free parameters, since both *m* and *N* in Eq. (3) are just positions in and lengths of the gene, which are known with certainty.

We can also use the model to predict the relative conversion frequencies of different positions, which can be measured experimentally. The exact Eq. (1) was used for analyzing the data, but for high ρ the relative conversion efficiency of positions *n*_1_ and *n*_2_ is well approximated by:

(4) $$\frac{P_{\text{eff}}(n_{1})}{P_{\text{eff}}(n_{2})}\approx \frac{n_{1}(N-n_{1})}{n_{2}(N-n_{2})}$$

This ratio only depends on the relative distances to the ends of the gene: if position *n*_1_ is 30% into the gene while position *n*_2_ is 90% into the gene, then position *n*_1_ is converted with a (0.3 × 0.7)/(0.9 × 0.1) ≈ 2.3 times higher frequency than position *n*_2_. Our experimental system allows us to estimate the conversion frequencies for the positions under selection, i.e., for the different values of *n* in Eq. (2).

### Conversion tract distributions in absence of MMR support simple model

**Figure 3:**
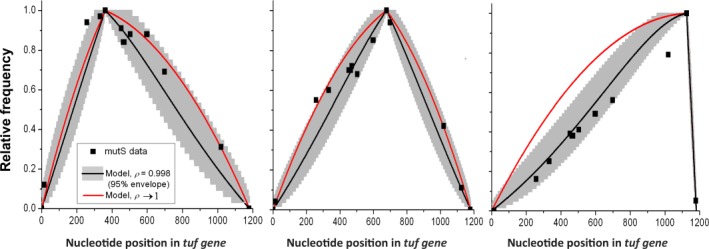
Frequency of conversion in mismatch repair‐deficient strains. Relative frequency of converted positions in mismatch repair‐deficient strains, for the three selection points 362 nt (left), 679 nt (middle) and 1126 nt (right), in a full‐length gene of *N* = 1185. The curves represent: experimental data (▪), the model in Eq. (1) for processivity parameter *ρ* = 0.998 corresponding to an average walk length of 500 nt in either direction (black line), and the limit distribution in Eq. (2) where the average walk length approaches infinity (red line). The grey envelopes are theoretical 95% confidence intervals given the binomial statistics for a two‐outcome process, using *ρ* = 0.998 and the experimental sample sizes of each experiment. Note that the errors of subsequent nucleotide positions are not independent: If an individual conversion event stops early, all downstream positions will be affected. The only slight anomaly from the predicted curve is observed for *n* = 1126 nt. However, calculating 95% confidence intervals from the same stochastic model, given the experimental sample size (*n* = 61), shows that some such deviations are statistically expected. Alternatively, the conversion rate at the selection position may be slightly reduced relative to the other positions due to minor local context effects from being too close to the end of the gene.

To analyze the kinetics of gene conversion without the confounding effects of subsequent MMR, we first compare the predicted distributions of conversion tract lengths to measurements in MMR‐deficient mutants. Strikingly, the simple model accurately reproduces the differently shaped distributions for all three of the selection points, for the same value of the single kinetic parameter ρ (Fig. 3). The best fit is observed for *ρ* ≈ 0.998, corresponding to an average unrestricted walk length of approximately 2/(1–*ρ*)=1000 nucleotides. We can statistically rule out a model fit both to *ρ* ≈ 0.999 and *ρ* ≈ 0.995, but also note that the limit distribution for ρ→1 still reproduces the basic shapes without depending on any parameters except the length of the gene and the position selected for, both of which are exactly known.

To further test the model we perturbed the gene‐specific parameters by studying a strain with a large transposon element inserted in one of the alleles. Specifically, an ∼11kb Mu*d*J transposon was inserted at nt 713 in the donor *tufB* gene, which greatly reduces the contiguous region of identity available for recombination and conversion of a mutant allele in *tufA* (nt 362). The results for this strain further confirmed that conversion is processive and blocked by large regions of sequence differences since all observed conversion tracts were on the same side of Mu*d*J as the selected position. We also found that the conversion tract distributions for this system were well captured by the same model for the same parameter value, *ρ* ≈ 0.998 (Supporting Information Fig. S2).

Finally, we tested the model for predicted conversion frequencies. In the MMR‐deficient strain, positions 362, 679 and 1126 were converted with approximate rates of 1 × 10^−3^, 2 × 10^−3^ and 2 × 10^−5^ per cell per generation (Table 1).

Because the rate of creating DSBs is unknown, we cannot predict absolute conversion frequencies, but the model does support the observation that the conversion frequency drops from the middle of the gene toward the ends, and that the effect is marginal except close to the ends. Specifically the model predicts that positions 362 and 1126 are converted with 86% and 17% of the conversion frequency of position 679 respectively. The difference between the predicted drop to 86% and the measured drop to 50% is well within the error of the measurements. For the position at 1126, the difference between the predicted drop to 17% and the measured 1% drop is statistically significant, and could suggest an effect of local context very close to the end of the gene, as also suggested by the conversion tract distributions. However, because selection point 1126 requires a different assay for measuring conversion rates, the poor quantitative fit is not necessarily reliable.

### MMR reduces the length of the conversion tracts and modifies their distributions

In MMR‐proficient strains most mismatched heteroduplexes will be detected by MutS, most likely resulting in destruction of the recombination event (Worth *et al*., 1994; Modrich and Lahue, 1996). By comparing the tract distributions with those obtained without MMR (Table 1, Fig. 3), we also pinpoint the effect of MMR.

**Figure 4:**
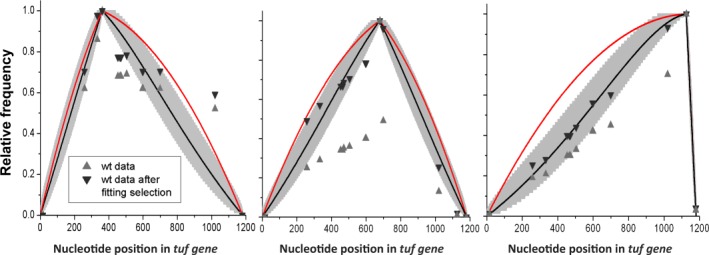
Frequency of converted positions in mismatch repair‐proficient strains. Relative frequency of converted positions in mismatch repair‐proficient strains, for the three selection points 362 nt (left), 679 nt (middle) and 1126 nt (right), in a full‐length gene of *N* = 1185. The curves represent: experimental data (▲), data with adjusted selection point (▼), the model in Eq. (1) for processivity parameter *ρ* = 0.998 corresponding to an average walk length of 500 nt in either direction (black line), and the limit distribution in Eq. (2) where the average walk length approaches infinity (red line). The 95% confidence envelopes were generated as in Fig. 2, using the sample sizes listed in Table 1. In the adjusted data set (▼), the percent conversion of the selection position was modified (and renormalized to one) to test if the rest of the data points approximately fit the same distributions as in the repair‐deficient strains.

The measurements show that active mismatch repair reduces the average length of final conversion tracts, but also changes the shapes of the distributions in various ways. For example, the position of the selected nucleotide (Fig. 4) is over‐represented compared with repair‐deficient strains, though if the selected nucleotide is discounted from the analysis, the rest of the distribution is approximately unchanged compared with the MMR‐deficient strains (Fig. 4). The most striking effect is seen for selection of nt 679, where 50% of conversion tracts have only the selected nucleotide converted and do not include either of the nearby flanking mismatched nucleotides. The great reduction in the rate of each of the three selected changes in *recA* strains rules out that this is caused by a high rate of spontaneous mutation (Abdulkarim and Hughes, 1996). A more likely explanation is that these anomalies are due to the different efficiencies with which MMR corrects different base‐base mismatches (Kramer *et al*., 1984). Specifically, the mismatch created at nt 679 will be either G/A or C/T depending on which DNA strands are paired (Figs 1 and 2), both of which are poorly repaired by MutS. The nearest mismatches that would be created on either side of nt 679 are A/C and T/G, each of which is recognized with high efficiency by MutS. This creates a situation where a short conversion tract including nt 679 but not the flanking nts 600 and 699 will be selected frequently because mismatch repair fails to detect and repair the selected mismatch. The same argument applies to conversion of nt 362 where the potential mismatches at the selected nucleotide (T/T or A/A) are repaired with low and intermediate efficiency, whereas the potential flanking mismatches at nts 333 and 453 (C/A and G/T) are both repaired with high efficiency (Figs 1 and 2). Thus, at nt 362 and nt 679, the relative efficiency with which MMR acts to repair different mismatches provides a plausible explanation for the observed anomaly in distribution of conversion tract lengths around the selected nucleotides. This explanation does not hold for the third selected position, nt 1126, where A/C or G/T, are each expected to be corrected with high efficiency. However, we note that selection for nt 1126 also produced an anomalous distribution in the MMR‐deficient *mutS* strain, arguing that the effect at this position is independent of the relative efficiency of MMR.

### Comparison of conversion frequencies in MMR‐proficient and MMR deficient strains also support the simple conversion model

Though we cannot measure directly the rate of forming DSBs, we can infer values that are consistent with the measured total rate of conversion and the estimated ρ≈0.998. With a total conversion rate of *r*_conv_ and a total gene length of *N*, the inferred rate per nucleotide of producing a DSB should follow *r*_DSB_ = *r*_conv_/(*N* × *P*_eff_) where *P*_eff_ from Eq. (1) takes into account that different starting positions have different probabilities of leading to successful conversion. For the two selection positions 362 and 679 (we omit position 1162 from the calculation due to the indications of deviations from the model close to the end of the gene), we measured *r*_conv_ of 1 × 10^−3^ and 2 × 10^−3^ respectively, and the model predicts that *P*_eff_ is 15% and 18% respectively. The two estimates for *r*_DSB_ are then 6 × 10^−6^ per nt and 9 × 10^−6^ per nt per cell per generation respectively.

The rate of gene conversion is greatly reduced (200–1000‐fold) in the MMR‐proficient strains (Table 1). This large difference suggests that creation of a heteroduplex region containing a mismatch that is recognized by MutS is a very frequent occurrence in the process in *tuf* gene conversion. In the MMR‐proficient strain, nt 679 is converted with rate 10^−5^ per cell and generation, and 50% of these convert only the selected nucleotide. This value of 5 × 10^−6^ also fits very well with the model: using ρ≈0.998, the probability that position 679 is converted without also converting either position 601 or 698, is 0.28% (using Eq. (1) with *N* = 98 and *m* = 79), and using the average estimated *r*_DSB_ of 7.5 × 10^−6^ per nt, then predicts a total rate of only observing conversion at position 679 in our experiments as 7.5 × 10^−6^ × 0.028 × 98 ≈ 2.1 × 10^−6^.

## Conclusion

From bacteria to humans, gene conversion acts as a cohesive evolutionary force by keeping repeated genes almost identical. Because many of those genes are central to growth, including highly expressed genes of the translation machinery, conversion could thus have substantial fitness consequences, whether by spreading advantageous mutations to other gene copies or by eliminating multiple slightly deleterious mutations in one swoop. Keeping repeated genes similar may even be advantageous in itself because other components and processes of the cell may co‐evolve more efficiently if they only have to accommodate a single version of an interacting protein.

**Figure 5:**
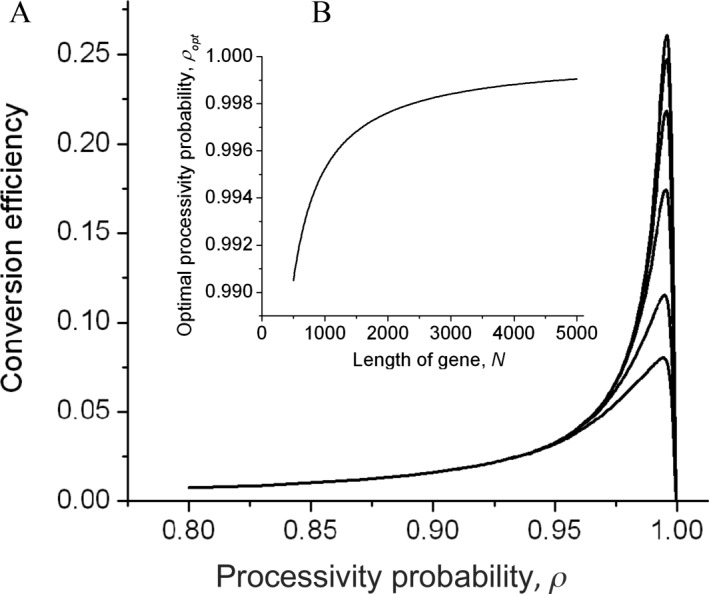
Conversion efficiency as a function of the processivity probability. A. The conversion efficiency (the probability that a random DSB results in a successfully converted position *m*) as a function of the processivity probability *ρ* in a gene of length *N* = 1185, for nucleotide positions *m* = [100 200 300 400 500 592]. By symmetry, the curves for *N* – *m* follow identically to the curves for *m*. Position 100 is thus converted at the same rate as position 1082. Position 592 corresponds to the middle of the gene. B. Insert zooms in on the parameter range of *ρ* > 0.99, and shows that the curves peak around the same value of *ρ*, which we denote *ρ*_opt_ This is the optimal processivity probability (the value of *ρ* for which the curves in Fig. 4a have a maximum), as a function of the total gene length, *N*. Note that for a typical length of 1000–2000 nts (see Discussion), *ρ*_opt_ is very close to the estimated value from Figs 3 and 4.

Cells that convert repeated genes efficiently are more likely to produce offspring that can take advantage of such changes. However, just as with mutation rates and other indirect determinants of fitness, it is unclear if this is sufficient to place the conversion mechanisms under effective selective pressure, or if they are simply by‐products of other processes such as DSB repair. By quantifying the process, our data may provide a clue to this problem. Specifically, the conversion efficiency depends on the rate of forming a DSB within the gene, the spreading of the conversion tract, and the probability of avoiding the mismatch repair system. Cells are under strong selective pressure to avoid DSBs and to efficiently repair mismatches, but the spread of the conversion tract could in principle be selected to maximize the conversion frequency. Specifically, the probability that a DSB leads to successful conversion is highest for some intermediate value *ρ*_opt_ of the processivity parameter, such that conversion tracts are not too short but also do not frequently extend to the ends of the gene where conversion aborts. Most repeated genes in *Salmonella* are in the range of 2–4 kb (Supporting Information, S3 text), and the model predicts that the processivity parameters that optimize the chance of successful conversion are $\rho_{\text{opt}}\approx 0.997$ and $\rho_{\text{opt}}\approx 0.999$ for 2kb and 4kb genes respectively (Fig. 5) – strikingly close to our experimental *in vivo* estimate of ρ≈0.998. This could reflect selection against potential cell death when conversions extend beyond the ends of the homologous sequences, since that risk increases greatly as ρ increases above 0.998. However, it certainly raises the possibility that gene conversion indeed evolved to maximize the efficiency with which repeated genes are kept similar to each other.

Our results showed that the simplest quantitative model – assuming uniform probabilities of initiation, progression and termination along the gene – can well explain the observed conversion rates and the nontrivial shapes of the conversion tract distributions. That does not rule out effects of local sequence on short length scales though. For example, if some nucleotide pair had much higher initiation or termination probabilities than other pairs, this would still only marginally affect the overall outcome since the different pairs appear so frequently along the gene whereas typical conversion tracts are quite long, smearing out repeated local effects. The same was true for the first empirical example of a homogenous Poisson process, observed for the number of Prussian army officers kicked to death by their horses: deaths appeared to arise with uniform probability over time, but given the long waiting time between events, that fit did not rule out differences on short time scales, such as brief refractory periods after each death or lower death probabilities at some time of day. In general, fits to simple uniform models only show that on the results are *effectively* as if the process was perfectly uniform, which is often surprising in itself. Specifically, given the potential complexity of the conversion process – initiation, elongation and termination could all be strongly dependent on rare sequences, many other processes could influence the outcome – we find it striking that the observations are so quantitatively captured by simply considering the lengths of the repeated sequence.

## Experimental procedures

### Bacterial strains and selections

Strains are derivatives of *S. enterica* serovar Typhimurium LT2 (Table 1). Identification of gene conversion at nt 362 in *tufA* (strains TH4498, TH5037, TH1193 and TH7511), and at nt 679 in *tufB* (strains TH522 and TH5038), was based on selection for fast growing colonies on LB agar (Fig. 2). Cells carrying conversions at nt positions 362 or 679 gave rise to easily distinguishable large colonies against a background of extremely small microcolonies 12–16 h after plating cultures on LB agar incubated at 37°C. In TH4498 and TH5037 the insertion of Mu*d*J, 17 nt upstream of the *tufB* coding sequence, silences *tufB* gene expression but leaves the entire *tufB* coding sequence available for recombination to convert nt 362 in *tufA*. In TH1193 and TH7511 Mu*d*J is inserted within the *tufB* coding sequence at nt 713, disrupting the *tufB* coding sequence and reducing the length of contiguous sequence homology available for recombination to convert nt 362 in *tufA*. Gene conversion at nt 1126 in *tufB* was selected by spreading cultures of TH488 or TH3262 on LC kirromycin plates to select kirromycin‐resistant colonies (Fig. 2). Resistance due to gene conversion was confirmed by DNA sequencing to identify the presence of the A375T mutation in both *tuf* genes (Abdulkarim and Hughes, 1996).

### Conversion rate measurements

Rates of *tuf* gene conversion per cell per generation were measured in fluctuation tests (Luria and Delbruck, 1943) and calculated as described previously (Abdulkarim and Hughes, 1996).

### Media

The complex medium LC with 2 mM EDTA (Abdulkarim and Hughes, 1996), was used in selections for kirromycin resistant mutants. Antibiotics were used at final concentrations as follows: 100 μg/ml kirromycin; 50 μg/ml kanamycin, 15 μg/ml tetracycline.

### PCR and DNA sequencing

Protocols and primers for amplifying and sequencing the *S. enterica tuf* genes have been described previously (Abdulkarim and Hughes, 1996).

## Conflict of Interest

The authors declare that they have no conflict of interest.

## Supporting information

Supporting Information
