# Supplementary material for: The processive kinetics of gene conversion in bacteria
Source: Mol Microbiol. 2017 Mar 13;104(5):752–60. doi: 10.1111/mmi.13661 (PMC5485169; doi:10.1111/mmi.13661)
Supplement: Supplementary file 1 — Supporting Information [file MMI-104-752-s001.pdf]

SUPPORTING INFORMATION

for *The processive kinetics of gene conversion*

by Paulsson, El Karoui, Lindell and Hughes

Here we derive the mathematical results in the main text, present the results for the MudJ insert in *tufB*, and discuss the average length of duplicated genes in *S. enterica*. The mathematical results were also double-checked by first-principle computer simulations (for details see below Eq. (S5))

S1 Text. Deriving the conversion probabilities

Here we implement the model above for a gene of arbitrary length N where i denotes the initial position of the DSB and n denotes one of the selection points. For the *tuf* gene, N =1185 and n = 362, 679 or 1126. The distribution of interest is the probability $\Pr(m|n)$ that the conversion tract contains position m given that it also contains position n. This can be calculated as $\Pr(m|n)=\Pr(m,n)/\Pr(n)$ where $\Pr(m,n)$ is the conditional probability that both positions m and n are present in the same conversion tract and $\Pr(n)$ is the probability that position n is present, regardless of m.

Because all steps are assumed independent, the probability Pr(m) that position m is successfully converted without proceeding beyond the end of the gene, is a product of three probabilities. For $i < n$, this is the probability of proceeding from i to n, multiplied by the probability of not proceeding from n to $N+1$, multiplied by the probability of not proceeding from position i to 0:

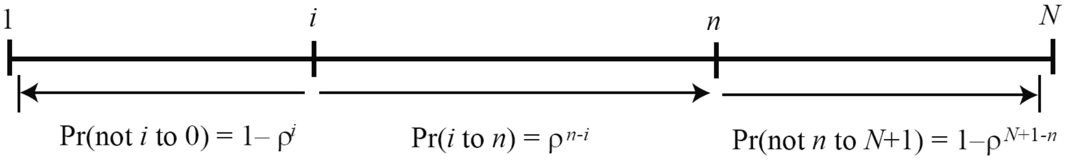

The sought probability is thus $\rho^{n-i}\left(1-\rho^{N+1-m}\right)\left(1-\rho^{i}\right)$ given that we start in position i. The probability that we start at position i is in turn $1/N$. Summing over all $i \le m$ gives

$$P\left(m|i\le m\right)=\frac{1}{m}\sum_{i=1}^{m}\rho^{m-i}\left(1-\rho^{N+1-m}\right)\left(1-\rho^{i}\right)=\frac{\rho^{m}-\rho^{N+1}}{N}\left(\frac{\rho^{-m}-1}{1-\rho}-m\right) \qquad \text{(S1)}$$

where $P\left(m|i<n\right)$ is the conditional probability that m is successfully converted given that the process starts to the left of m. The same procedure for $i > m$ gives

$$\begin{aligned}P\left(m|i>m\right)&=\frac{1}{N-m}\sum_{i=m+1}^{N}\left(1-\rho^{m}\right)\rho^{i-m}\left(1-\rho^{N+1-i}\right)\\&=\frac{\rho^{-m}-1}{N}\left[\frac{\rho}{1-\rho}\left(\rho^{m}-\rho^{N}\right)-\rho^{N+1}\left(N-m\right)\right]\end{aligned} \qquad \text{(S2)}$$

The total probability is

$$P_{eff}\left(m\right)=P\left(m\right)=\frac{m}{N}P\left(m|i\le m\right)+\frac{N-m}{N}P\left(m|i>m\right) \qquad \text{(S3)}$$

where m/N and $(N-m)/N$ are the probabilities that a certain DSB were in the intervals $1\le i<m$ and $1<m\le N$. Combining Eqs. (S1)-(S3) generates Eq. (1).

The joint probability $P(m,n)$ that both positions *m* and *n* are present in the same conversion tract, can be calculated assuming $m < n$ with no loss in generality. The calculation is broken up into three parts, depending on the position of the DSB:

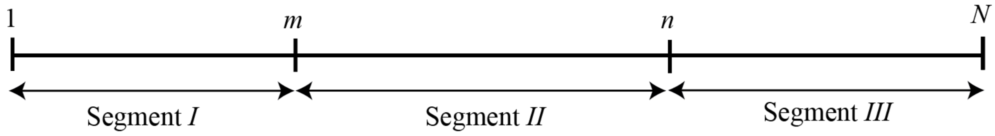

The contributions from the three segments then follow

$$I:\ \frac{1}{N}\sum_{i=1}^{m}\underbrace{\rho^{n-i}}_{\text{Include } n}\left(\underbrace{1-\rho^{N+1-n}}_{\text{Not include } N+1}\right)\left(\underbrace{1-\rho^{i}}_{\text{Not include } 0}\right)=\frac{\rho^{n}-\rho^{N+1}}{N}\left(\frac{\rho^{-m}-1}{1-\rho}-m\right)$$

$$II:\ \frac{1}{N}\sum_{i=m+1}^{n}\underbrace{\rho^{n-i}}_{\text{Include } n}\ \underbrace{\rho^{i-m}}_{\text{Include m}}\left(\underbrace{1-\rho^{N-n}}_{\text{Not include N+1}}\right)\left(\underbrace{1-\rho^{m}}_{\text{Not include } 0}\right)=\frac{\rho^{n}-\rho^{N}}{N}\left(\rho^{-m}-1\right)\left(n-m\right) \tag{S4}$$

$$III:\ \frac{1}{N}\sum_{i=n+1}^{N}\underbrace{\rho^{i-m}}_{\text{Include m}}\left(\underbrace{1-\rho^{N-i}}_{\text{Not include N+1}}\right)\left(\underbrace{1-\rho^{m}}_{\text{Not include } 0}\right)=\frac{\rho^{-m}-1}{N}\left[\frac{\rho}{1-\rho}\left(\rho^{n}-\rho^{N}\right)-\rho^{N}\left(N-n\right)\right]$$

and the total probability sums to

$$\Pr(m,n)=\frac{p^{n}-p^{N}}{N}\left[\left(n-m\right)p^{-m}+\left[\frac{1+p}{1-p}-\frac{p^{N}\left(N-n\right)}{p^{n}-p^{N}}\right]\left(p^{-m}-1\right)-n\right] \tag{S5}$$

The conditional probability in Eq. (2) then directly follows from $\Pr(m|n)=\Pr(m,n)/\Pr(n)$.

To ensure the correctness of the derivations, we also designed a straightforward first-principle Monte-Carlo simulation based on the same assumptions. In each round of the simulation, we initiate a single DBS with uniform probability at each position in the gene. We then simulate conversion to the left and right by drawing uniform random numbers and proceeding with the conversion with probability ρ. Each resulting conversion tract is stored, and the process is iterated at least 10^6 times. Finally, we discard tracts that proceeded beyond either end of the gene, or that do not include the selection position. Each simulation was then repeated for each of the three selection points and a very wide range of ρ-values.

The simulation results were identical to the analytical solutions down to the miniscule error at a sampling of 10^6 simulations for all parameters tested: for all the figures of the paper, the simulated curves cannot be separated from the analytically derived curves. They also perfectly agree on the expected sampling errors, included in the theoretical confidence intervals. However, we also note that the agreement does not lend any further support to the model – because the two approaches start with the same assumption, the simulations must converge to exact analytical distributions unless there are errors in either approach. The simulations are instead done to convince readers who do not wish to go through all the algebraic steps of the derivations, and to reduce the risk of errors in the derivations.

SUPPORTING INFORMATION

for *The processive kinetics of gene conversion*

by Paulsson, El Karoui, Lindell and Hughes

S2 Figure. Distribution of conversion tracts for the MudJ transposon.

We selected for conversion at nt 362 using a MMR deficient strain in which a ~11kb Mu*d*J transposon is inserted at nt 713 in the donor *tuf* gene. This tested for consistency with the model, and the results are shown below.

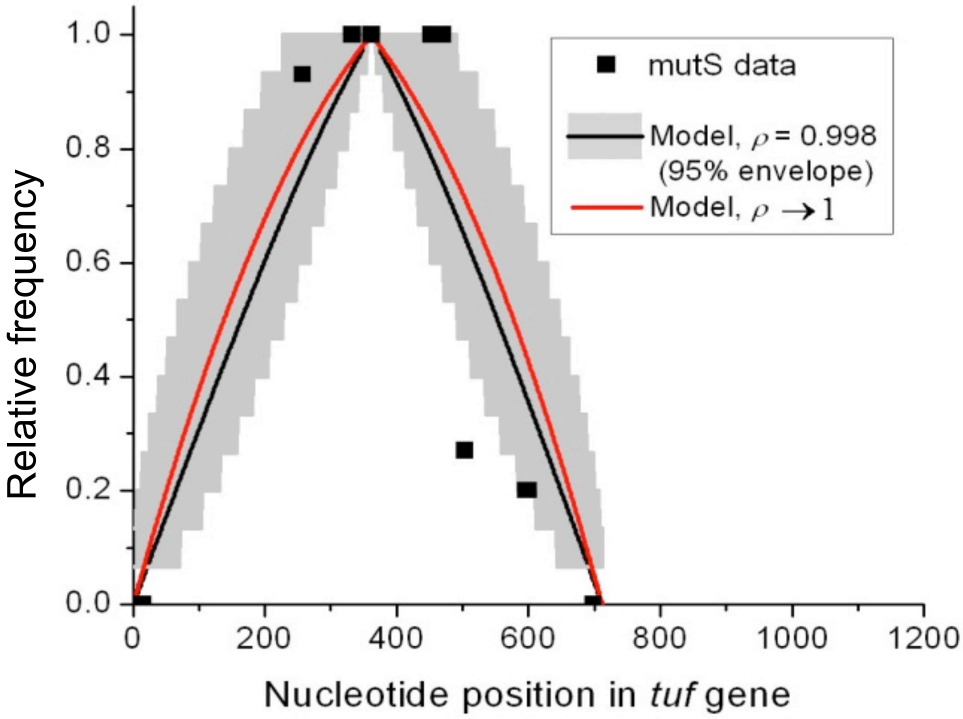

Figure S2. Relative frequency of converted positions in repair-deficient strains, selecting for nt position 361 and using a MudJ transposon in nt position 713 of the donor *tuf* gene. The curves represent: experimental data (■), the model in Eq. (1) of the main text for processivity parameter ρ = 0.998 (black line), and the limit distribution in Eq. (2) of the main text where the average walk length approaches infinity (red line). The grey envelopes are theoretical 95% confidence intervals given the binomial statistics for a two-outcome process, as described above in S1 and in Fig. 2 of the main text, using ρ = 0.998 and the experimental sample size of the experiment (n=40).

SUPPORTING INFORMATION

for *The processive kinetics of gene conversion*

by Paulsson, El Karoui, Lindell and Hughes

Here we discuss the average length of duplicated genes in *S. enterica*. The mathematical results were also double-checked by first-principle computer simulations (for details see below Eq. (S5))

S3 Text. Average length of duplicated genes in *Salmonella*

The main repeated coding sequences in *Salmonella* (not counting IS elements) are:

- Two copies of the *ccm* operon, 7.5 kb, 99% nt identity.
- Seven copies of *rrs* (16S rRNA) 1541-1544 nts, 99-100% nt identity.
- Five copies of *rrl* (23S rRNA) 2993 nts, 99-100% nt identity. (There is also a 3009 nt copy with one intervening sequence, and a 3092 nt copy with 2 intervening sequences).
- Seven copies of *rrf* (5S rRNA) 119 - 121 nts, 7 copies. The *rrn* genes below have about 99-100% nt identity
- Two copies of a duplicated region in *pagJ* and *pagK*, 1688 nts, 90% identity at nt level.
- Two copies of *tuf* genes, 1185 nts. 99% nt identity

The *ccm* operon is concerned with cytochrome C biogenesis and is the longest duplicate sequence in the *Salmonella* genome. The *pag* genes are PhoP-PhoQ activated. All other genes (mostly in the *rrn* ribosomal group) are involved in translation.

Longer genes have proportionally more sites for double-stranded breaks, and should perhaps therefore be counted proportionally more towards the average. Weighting each gene by its length relative to the average is equivalent to accounting for the variance in length σ_L^2. With L_i as the length of duplicated sequence number *i*, the un-weighted and weighted average are then:

$$\langle L \rangle = \frac{L_1 + L_2 + ...L_n}{n}$$

$$\langle L \rangle_{weighted} = \frac{L_1 \times \frac{L_1}{\langle L \rangle} + L_2 \times \frac{L_2}{\langle L \rangle} + ...L_n \times \frac{L_n}{\langle L \rangle}}{n} = \frac{\langle L^2 \rangle}{\langle L \rangle} = \langle L \rangle \left(1 + \frac{\sigma_L^2}{\langle L \rangle^2} \right) \qquad (6)$$

Including all genes above, except the *rrl* genes with intervening sequences, we have $\langle L \rangle = 1.9\text{kb}$ and $\langle L \rangle_{weighted} = 3.9\text{kb}$.
